# Mechanisms of Resistance to Chemotherapy in Breast Cancer and Possible Targets in Drug Delivery Systems

**DOI:** 10.3390/pharmaceutics12121193

**Published:** 2020-12-09

**Authors:** Patrícia de Faria Lainetti, Antonio Fernando Leis-Filho, Renee Laufer-Amorim, Alexandre Battazza, Carlos Eduardo Fonseca-Alves

**Affiliations:** 1 Department of Veterinary Surgery and Animal Reproduction, Sao Paulo State University–UNESP, Botucatu-SP 18618-681, Brazil; patricia.lainetti@unesp.br (P.d.F.L.); a.leis@unesp.br (A.F.L.-F.); 2 Department of Veterinary Clinic, Sao Paulo State University–UNESP, Botucatu-SP 18618-681, Brazil; renee-laufer.amorim@unesp.br; 3 Department of Pathology, Botucatu Medical School, São Paulo State University–UNESP, Botucatu-SP 18618-681, Brazil; alexandre.battazza25@unesp.br; 4 Institute of Health Sciences, Paulista University–UNIP, Bauru-SP 17048-290, Brazil

**Keywords:** neoadjuvant therapy, treatment resistance, siRNA nanoparticles, liposomal nanostructures

## Abstract

Breast cancer (BC) is one of the most important cancers worldwide, and usually, chemotherapy can be used in an integrative approach. Usually, chemotherapy treatment is performed in association with surgery, radiation or hormone therapy, providing an increased outcome to patients. However, tumors can develop resistance to different drugs, progressing for a more aggressive phenotype. In this scenario, the use of nanocarriers could help to defeat tumor cell resistance, providing a new therapeutic perspective for patients. Thus, this systematic review aims to bring the molecular mechanisms involved in BC chemoresistance and extract from the previous literature information regarding the use of nanoparticles as potential treatment for chemoresistant breast cancer.

## 1. Introduction

Breast Cancer (BC) is one of the most important cancers in women worldwide, according to the last global cancer statistics, and it was the second-leading cause of cancer-related deaths in 2018 [1]. Chemotherapy is seldom used for treating BC, but in specific cases, it may be recommended [2]. Usually, BC is classified into molecular subtypes, and for some of them, chemotherapy is an option. Among the molecular subtypes, triple-negative BC is considered one of the most aggressive, and its chemotherapy response rate is considered higher when compared to the others. However, despite adjuvant chemotherapy, the overall survival of these patients is still poor [3].

Since chemotherapy is usually used for triple-negative, inflammatory and advanced-stage BC, new strategies and molecular predictive markers are required to increase the patient’s prognosis [4]. New predictive and prognostic markers can provide valuable information regarding the identification of patients that could benefit from chemotherapy. Besides that, different strategies can be used to increase drug delivery into tumor cells, including nanoparticles. Different systems of nanostructured carriers can be effective in cancer chemotherapy and overcome drug resistance. In this scenario, this manuscript aimed to critically review the previous literature regarding BC chemoresistance, elucidating its molecular features and providing the perspective of nanocarrier structure use to reduce tumor chemoresistance.

## 2. Breast Cancer

Cancer is a common disease, globally distributed and with over 60% growth rate expected for the next 20 years [1]. Among the most common types of cancer, BC is the second-most frequently diagnosed, only after lung tumors [1]. In women, it is the most common type of cancer, representing about 30% of new cases [5]. In 2018, more than two million women were affected by this disease, with a mortality rate of 6.6%, resulting in more than 500 thousand deaths of women [1]. There has been a 40% decline in mortality from breast tumors with the evolution of diagnostic tools and treatments, but since 2010, the reduction in the mortality rate has slowed [5].

The definitive diagnosis is made by histopathology, and tumors should be classified according to Lakhani et al. [6]. For the staging system, the patient’s tumor size, nodes and metastasis occurrence are evaluated according to Giuliano et al. [7]. The most prevalent subtypes of breast cancer are invasive carcinoma (70–75%) and lobular carcinoma (12–15%) [8]. Each histological subtype has a specific prognosis and treatment protocol [7,8]. BC can also be classified into molecular subtypes according to their expression of estrogen receptors (ER), progesterone receptors (PR) and human epidermal growth factor (HER2). Moreover, Ki67 is a marker used to estimate tumor proliferation and chemosensitivity and, also, have some prognostic value for certain molecular subtypes [9,10]. Thus, the patient’s diagnosis and prognosis are made based on a multidisciplinary approach.

Tumors can be divided into a luminal A-like subtype (ER+ and/or PR+ and HER2-, low Ki67 index); luminal B-like subtype HER2+ (ER+ and/or PR+ and HER2+, high Ki67 index) or luminal B-like HER2- (ER+ and/or PR+ and HER2-, high Ki67 index); HER2 subtype (ER-, PR- and HER2 +, high Ki67 index) and triple-negative (ER-, PR- and HER2-, high Ki67 index) [9,10,11]. Luminal A tumors are the most common molecular subtype of breast carcinoma (30–40%), followed by luminal b (20–30%), HER2 overexpressed (12–20%) and triple-negative (10–15%) [11,12].

Tumors classified as luminal A are associated with a favorable prognosis and, usually, low-grade luminal B with an unfavorable prognosis, higher tumor grades and proliferative activity; HER2 are overexpressed with a higher incidence of local or regional recurrences and triple-negative with a poor prognosis, higher rates of recurrence, distant metastases and mortality [11,12]. Triple-negative tumors are considered the most challenging molecular subtype, once treatment options are scarce, because they have more aggressive and metastatic behavior, resulting in a shorter disease-free interval and survival time for patients [13]. In addition, malignant breast neoplasms are the major condition leading to the high mortality of women. For these reasons, prevention and new therapies are essential for a better prognosis [14].

## 3. Neoadjuvant Chemotherapy for Breast Cancer

In general, BC treatments focus on the cure of the disease, higher disease-free survival (DFS) and overall survival (OS) time and quality of life. Different types of modalities can be associated, such as local, systemic and support treatments. Local treatments include surgery and radiotherapy, and systemic therapies can be divided into chemotherapy, immunotherapy and target and hormone therapy [8,15]. The choice of the treatment must consider the tumor location and size, lymph node commitment, histopathology, molecular subtype and presence of metastases. Moreover, the patient’s health condition, age, hormonal status and preferences should be also discussed [8].

In patients with nonmetastatic tumors, it is recommended to perform local therapy, with surgical removal of the tumor and, in some cases, of the axillary lymph nodes with subsequent radiotherapy. Radiation therapy is recommended for most patients after local surgery with breast conservation. In addition, systemic therapy can be used as a neoadjuvant and/or adjuvant tool for surgery [16,17]. In luminal tumors A and B, tumors ER+ and HER2-, the standard treatment is with adjuvant endocrine therapy. Some patients with this type of tumor can have some benefits adding chemotherapy. Women with stage III tumors and patients with commitments of four or more lymph nodes, even if it is a lobular carcinoma and/or grade 1 tumor or luminal A, should receive chemotherapy [16]. HER2+ tumors should be treated with chemotherapy and targeted monoclonal antibody therapy, and triple-negative tumors are treated mainly with chemotherapy [16,17].

Alkylator and taxane-based regimens with anthracycline are chemotherapy drugs recommended for Luminal A and B BCs. HER2+ tumors, in stage 2 or 3, should be treated with anthracycline-, alkylator- and taxane-based chemotherapy in combination with trastuzumab or pertuzumab and stage 1 with paclitaxel and trastuzumab [16]. The most used treatments for triple-negative breast cancer in women are doxorubicin in combination with cyclophosphamide, doxorubicin with cyclophosphamide and paclitaxel, and in cases of recurrence and resistance to doxorubicin, docetaxel can be used together with cyclophosphamide [17,18,19]. In patients with triple-negative tumors and metastasis at the time of diagnosis, the first line of treatment is chemotherapy with taxanes (paclitaxel and docetaxel), platinum compounds (cisplatin) or anthracyclines (doxorubicin) as a monotherapy [17,18,20].

Triple-negative BC treatment is based on the molecular characteristics of the tumor, and some proposed treatments are chemotherapy with anthracyclines, taxanes, alkylators and platinum-based, PARP1 inhibition when the tumor has an absence of or reduced BRCA1 function, antibody treatment when the tumor overexpresses Epidermal Growth Factor Receptor (EGFR), c-KIT tyrosine kinase inhibitor when it overexpresses c-KIT and multikinase inhibitors when overexpressing EGFR [4,16,19]. Metastatic breast tumors are treated like nonmetastatic breast tumors; however, the focus is on prolonging the patient’s survival using palliative therapy [17].

Triple-negative tumors do not have specific targets for therapies, nor do they have estrogen and progesterone receptors for hormone therapy. Thus, treatment options are more restricted. Therefore, even in cases without metastasis to distant organs or lymph nodes, chemotherapy is always used. In the case of triple-negative breast tumors with metastases, other treatment options can be used, in conjunction with chemotherapy, such as immunotherapy and PARP inhibitors and cisplatin or carboplatin [13].

## 4. Breast Cancer Molecular Mechanisms of Chemoresistance

**Figure 1:**
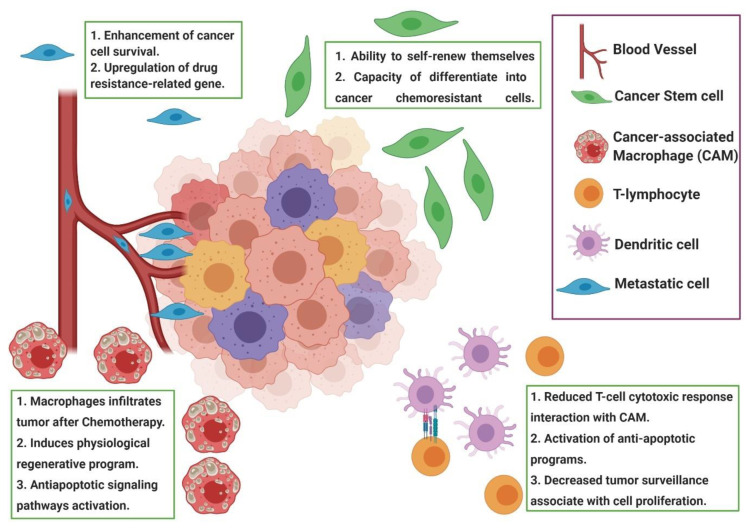
Representation of the tumor microenvironment factors associated with chemoresistance. During chemotherapy, the tumor microenvironment shows cancer-associated macrophages (CAM), cancer stem cells and other immune cells that induce paracrine signaling, leading to tumor resistance induced by drugs. Usually, the modulation of the tumor microenvironment after chemotherapy induces the recruitment of CAM and a reduced cytotoxic response of T cells, leading to a resistance to apoptosis. Figure created in BioRender (https://biorender.com/).

**Figure 2:**
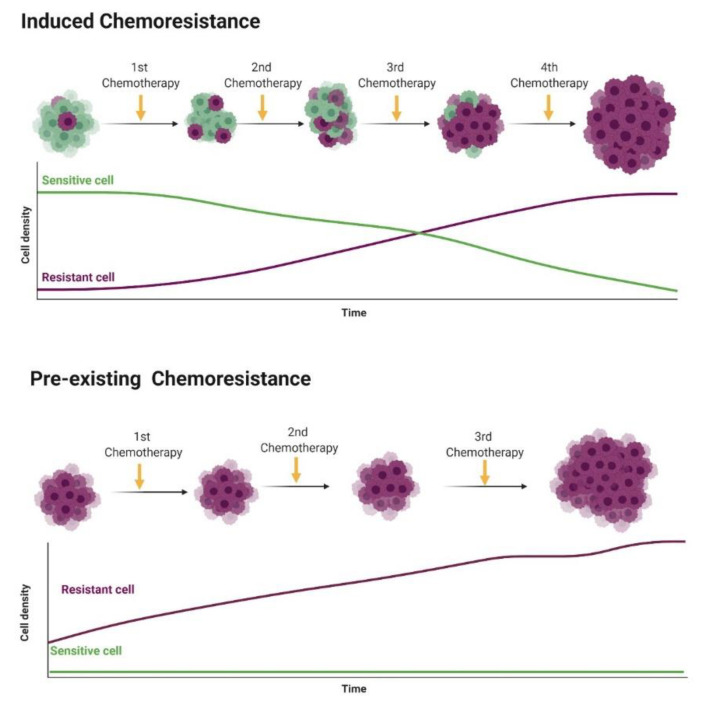
Representation of intrinsic chemoresistance in breast cancer (BC). Usually, tumor cells show individual genetic and epigenetic alterations that lead to chemoresistance (purple cells). In the induced chemoresistance, tumor cells are sensitive to chemotherapy, and during treatment different factors (such as hypoxia, oxidative stress and DNA damage) induce a selection of chemoresistant cells that grow after the apoptosis of sensitive cells by chemotherapy. Then, in a late stage, only chemoresistant cells are growing in the tumor microenvironment. On the other hand, in the pre-existing chemoresistance, cancer cells at the beginning of the therapy show chemoresistance and no response to therapy (cells naturally chemoresistant). Figure created in BioRender (https://biorender.com/).

Chemoresistance or drug resistance is described as the low efficiency and efficacy of a drug to produce a beneficial response in the treatment and, also, is one of the main factors associated with chemotherapy failure [21,22,23]. Tumor chemoresistance can be associated with different mechanisms, including interactions among cancer cells and the tumor microenvironment (Figure 1), cancer cell heterogeneity, cancer stem cells, cancer-associated macrophages and immune cells modulation, can modify the tumor microenvironment during chemotherapy, leading to chemoresistance. Chemoresistance can have intrinsic factors, like tumor heterogeneity, cancer stem cells and epigenetics and/or extrinsic/acquired factors (Figure 2), that includes pH, hypoxia and paracrine signaling and other tumor cells [22,24]. Cell resistance mechanisms in breast cancer involve cell membrane drug absorption, transport and efflux, transporter proteins, cancer-related genes (oncogenes and tumor-suppressor genes), DNA repair, cancer steam cells, the tumor microenvironment and epithelial-mesenchymal transitions (EMT) [22,25].

Chemotherapy resistance is an obstacle for the treatment of neoplasms and compromises the choice of chemotherapy for cases of recurrence. Therefore, it is increasingly important to choose an accurate and effective treatment, in a more personalized way, for each patient. Polychemotherapy or adjuvant therapies to conventional chemotherapy have been used more frequently, due to the heterogeneity of neoplasms and their genomic map [26]. When tumors become resistant to conventional therapy, new types of treatments are necessary.

### 4.1. Multidrug Resistance (MDR)

Cells that are capable of surviving after chemotherapy treatment usually have a multidrug resistance (MDR) ability. A MDR system promotes the efflux of anticancer drugs from tumor cells, reducing drug absorption. MDR can explain the resistance from some tumor cells to chemotherapy and depend on membrane transporters protein activity [22,24,27].

ATP-binding cassette (ABC) transporters can be found on the plasma membranes of cells and cellular vesicles and are known for transporting different molecules across cell membranes using energy from ATP and, also, as the largest family of transmembrane proteins [24,28].

P-glycoprotein (or ABCB1/MDR1) participates in the substance efflux through cell membranes and is part of the ABC transporter family. This protein can bind to some chemotherapy drugs, such as anthracyclines, taxanes and mitoxantrone, and remove them from the cell interior and release them into the extracellular space [22,25,27]. Breast cancer resistance protein (ABCG2/BCRP) is known to transport 5-Fluorouracil, methotrexate, doxorubicin, irinotecan, mitoxantrone and other drugs to outside of the cell [25]. ABCG2 is related to breast cancer cell resistance to doxorubicin when overexpressed [29]. MDR-associated proteins (MRPs) promote a drug excretion from the tumor cells and change the intracellular drug distribution [25,30].

Increased expression of the ABC transporter genes can cause resistance to drugs used in cancer treatments. *MDR1* gene expression, P-glycoprotein and alpha estrogen receptor (ERα) in breast tumors can induce chemotherapy resistance by promoting the efflux of chemotherapy drugs from neoplastic cells and by the activating DNA methylation [31].

### 4.2. Cancer Stem Cells

Cancer stem cells (CSCs) are a part of the population of tumor cells, which have the ability to form the tumor, self-regenerate, to multiple differentiate, are resistant to chemotherapeutic drugs, help tumor growth and are responsible for tumor recurrences [30]. CSCs are responsible for tumor heterogeneity, which is fundamental for tumor survival and its invasiveness [32].

These cells behave similarly to stem cells and have a long life due to the mechanisms of resistance to drugs and toxins, DNA repair capacity and resistance to apoptosis. They manage to survive chemotherapy treatments or metastasize to distant organs and cause tumor recurrence [22]; for these reasons, they have an important role in tumor resistance. In addition, they overexpress the different types of ABC transporters [25].

Usually, CD24, CD44, CD47, CD133, CD166 and ALDH1 expressions are usually used as CSC surface markers for BC [25,32]. Markers such as CD 44 and CD 24 are related to tumor behavior and a high capacity for invasion, migration and proliferation, respectively [32], and ALDH1 with a worse prognosis [25]. Thus, several studies have associated the expression of these markers by cancer cells as a negative prognostic factor and, also, associated with tumor chemoresistance [25].

### 4.3. Signaling Pathways

Signaling pathways are also associated to cell resistance, survival, growth and invasion [25,27]. The PI3K/AKT/mTOR and RAS/MAPK/ERK pathways are related to the resistance to endocrine treatments in breast tumors [25] and the PI3K/AKT/mTOR pathway always activated in HER2+ tumors [33]. The PI3K/AKT/mTOR pathway is related to the regulation of tumor cell apoptosis, tumor aggressiveness and a worse prognosis [27]. The JAK/STAT pathway is linked to the malignant process of tumor cells, tumorigenesis, survival, proliferation, angiogenesis and metastasis [27,34]. The RAS/MAPK/ERK pathway acts on cell proliferation and survival [28].

The presence of mutations in genes like *BRCA1*, *BRCA2*, *TP53*, *PTEN*, *CDH1*, *STK11*, *PALB2*, *JAK2* and *HIF1A* are related to an increased risk of developing breast cancer [35,36]. In addition, the presence of *AKT1*, *TP53*, *KDR*, *c-KIT*, *BRCA1* and *BRCA2* mutations are correlated with a poor prognosis, and, specifically, the mutation in the *PIK3* is linked to the poor prognosis of triple-negative tumors [37]. It has been shown that patients with allele changes and losses in the *BRCA1*, *RAD51B*, *PALB2* and *ERCC* genes have breast tumors resistant to chemotherapy [38]. Changes such as amplifications or mutations in *ESR1*, *PIK3CA*, *FGFR1*, *CCND1*, *TP53*, *MYC* and *ERBB2* genes have been associated with breast tumor chemoresistance in women [39].

Some studies have already demonstrated the positive relationship between genetic mutations and chemoresistance. The changes in loss or gain and/or mutations of the alleles have already been related to survival time, disease-free time, chemosensitivity and chemoresistance [38,39,40,41]. The polymorphisms found in the *ERBB3* and *BARD1* genes have demonstrated that patients with breast tumors presenting these alterations were more prone to recurrences and did not respond to treatment with polychemotherapy when compared to patients without these alterations [40].

### 4.4. Epithelial-Mesenchymal Transition

Epithelial-mesenchymal transition (EMT) is a mechanism in which epithelial cells lose their polarity and adhesion and acquire invasive and migratory properties, such as mesenchymal cells. There is a change in the epithelial cell phenotype, which occurs due to protein changes and transcriptional events in response to extracellular stimuli, that can be reversible or not [30]. The E-cadherin gene (*CDH1*) is highly expressed in epithelial cells and poorly expressed in mesenchymal cells and is one of the main participants in the EMT process [28]. When its function or expression is reduced, it allows noninvasive cells to become invasive, thus having a very important role in the invasion capacity of the tumor and metastasis [25]. Breast tumor cells with a mesenchymal phenotype have a higher capacity to develop resistance to chemotherapy drugs and are also associated with CSCs [28].

### 4.5. Tumor Microenvironment

The tumor microenvironment is composed of several cells with different phenotypes and genotypes, corroborating to the tumor heterogeneity, in addition to several proteins, such as cytokines and growth factors, the extracellular matrix, other cell types and a large number of blood vessels that supply the tumor. The microenvironment can interfere with how the tumor reacts to chemotherapy [22,24,25].

Most tumors have a microenvironment with little oxygen due to the fast consumption of tumor cells because of their high proliferation rate and an insufficient amount of blood vessels, mainly in the center of the tumor, which do not provide the ideal amount of oxygen for the large number of cells with a high cell proliferation rate [28]. Oxygen deficiency is related to the chemotherapy resistance activation genes, leading to the proliferation of cells resistant to hypoxia. Chemotherapy also has its action reduced in low oxygen environments [22].

The pH of the microenvironment also interferes with the antitumor drug’s effectiveness, cell proliferation, metastasis and tumor resistance. If the environment is acidic, some drugs may not be transported correctly and their action is impaired, and if it is alkaline, they may increase the cytotoxicity of some drugs [22,25]. Hypoxia helps to make the tumor microenvironment more acidic, also leading to cell resistance to chemotherapy drugs [27].

## 5. Epigenetics and Breast Cancer Chemoresistance

Endocrine therapy is one of the first-line treatments for BC, since two-thirds of the cases express an estrogen receptor (ER) [42]. It has been successfully used for luminal BC patients, with significant increased overall survival [43]. However, cancer cell resistance to endocrine therapy can develop, and some of the patients may experience tumor recurrence [43]. Interestingly, the mechanisms involved in resistance to endocrine therapy usually involve the epigenetic machinery. In the ER-positive cell line, ESR1-promoter hypermethylation was previously associated with ER downregulation and resistance to endocrine therapy [44,45]. Besides that, the transcriptional ER regulation may impact directly on the PR expression, and the parts of the tumor that are positive for ER also express PR [46]. The PR gene presents a CpG island in its first exon, and around 40% of RP-negative BC are associated with RP-promoter hypermethylation [47]. Recently, using a BC cell line model, it was demonstrated that, in a hormone-free cancer cell, when a decreased PR expression is induced, *ESR1* also showed a decreased expression associated with a hypermethylation of the *ESR1* promoter [48]. Thus, the ER and PR seem to be regulated by methylation in human BC cell lines, and also, methylation seems to promote a gene-cross regulation. Thus, new therapeutical strategies focused on epigenetic modifications can be associated with patients in hormone-negative BC patients.

Fibroblast growth factor receptor 1 (FGFR1) has been linked to the progression, proliferation, migration and survival of breast tumor cells [49]. It can be considered a predictive factor for decreasing patient survival in HR+ tumors and confers a resistance to estrogen. *FGFR1* amplification is related to a resistance to ER, PI3K and CDK4/6 inhibitors, providing resistance to different drug therapies [49]. A high FGFR1 expression has also been linked to a worse prognosis and overall survival in cases of triple-negative tumors and is a significant gene for tumor cell survival, with inhibition of the FGFR pathway being an adjunct therapy to chemotherapy and, also, an alternative to cancers refractory to treatment [50,51].

Another member of the fibroblast growth factor family, FGF-2, is related to chemoresistance in triple-negative breast tumors. Its super-expression helps to regulate the cell cycle and survival, in addition to assisting resistance to radiotherapy and metastasis formation [52]. FGF-2 overexpression has been reported in subpopulations of chemoresistant cells, leading to the formation of metastases and a worse prognosis [52]. In addition, when targeted for treatment and downregulated, the cell apoptosis and chemotherapeutic sensitivity of chemically resistant cells increases [53].

The *MAPK8* gene, also known as *JNK1*, is related to tumor cell survival by controlling autophagy by phosphorylation of the antiapoptotic protein BCL-2 [54]. JNK1 is associated with the chemoresistance of tumor cells, and its expression stimulates the overexpression of P-glycoprotein, conferring resistance to doxorubicin. Blocking the JNK or MAPK pathway decreases the expression of the ABCG2 transporter and leads to tumor cell autophagy [54,55].

Besides gene methylation, microRNA dysregulation has been implied in BC chemoresistance [56,57]. When a microRNA targets a tumor-suppressor gene, its overexpression is expected and induces gene downregulation [50]. In BC patients, miR-27b is associated with tumor invasiveness and migration [58], and miR-200a [59] and miR-155 [60] were previously related to apoptosis inhibition. Since chemotherapy induces cellular apoptosis, miR-200a and miR-155 overexpression can lead with BC chemoresistance. Interestingly, microRNA overexpression can be a therapeutic target, and a combination of drug delivery systems with a microRNA inhibitor can increase drug delivery and enhance the antitumor response. Wang et al. (56) identified several microRNAs associated with BC resistance using bioinformatics tools and extracted transcriptome data from drug-resistant BC patients (*N* = 5) compared to drug-sensitive BC patients (*N* = 5). In this analysis, 22 dysregulated microRNAs were identified, 12 downregulated and 10 upregulated (Table 1). Among the dysregulated microRNAs, hsa-miR-195a-5p showed the highest fold change (FC: 5.44) and hsa-miR-4472 the lowest fold change.

**Figure 3:**
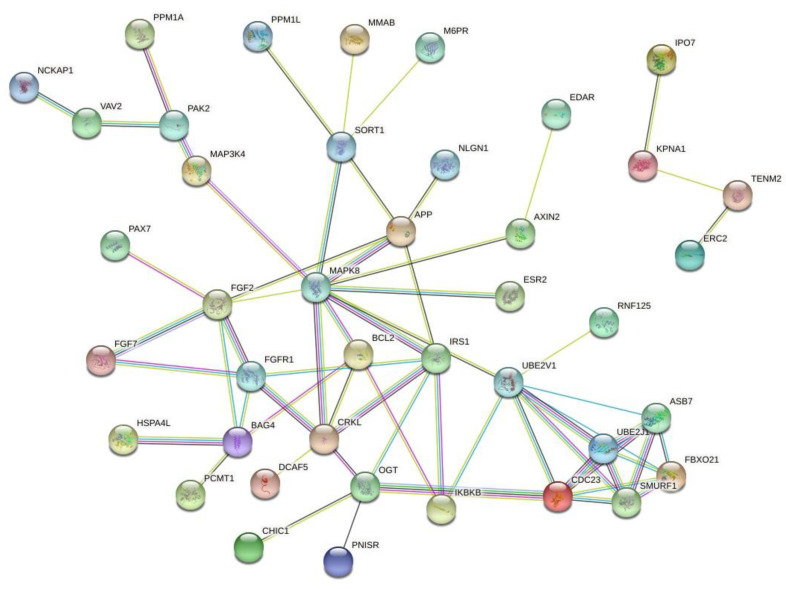
Protein-to-protein interaction (PPI) network based on the prediction of genes regulated by hsa-miR-195a-5p. Since this microRNA was associated with cancer chemoresistance, these genes may represent important drug targets. Figure generated with the online tool STRING (https://string-db.org/).

Since small-interfering RNAs (siRNAs) can be associated with chemoresistance, it is important to identify the target genes regulated by these long noncoding RNAs and, also, identify the interactions among the target genes. For this reason, we performed a search on the TargetScanHuman database (http://www.targetscan.org/vert_72/) to identify the hsa-miR-195a-5p target genes, and we identified 127 genes. Then, we performed a protein-to-protein interaction (PPI) prediction using the STRING database (https://string-db.org/), and we identified two major PPI networks. We identified two major networks, and to increase the visibility, we excluded the disconnect nodes and presented the two networks in Figure 3. Among the genes, we identified *FGF2*, *FGFR1* and *MAPK8*. These genes are regulated by hsa-miR-195a-5p, and they may associate with tumor chemoresistance and can be used as future targets to new drugs.

## 6. Markers Associated with the Chemotherapy Response

Genetic markers have been explored as a predictor of the antitumor response or chemoresistance, and the use of next-generation sequencing provided a new perspective for marker identification. Several markers were previously associated with BC drug sensitivity or resistance [61,62,63,64,65,66,67,68,69]. Gong et al. [62] evaluated a cohort of 421 patients with luminal A BC from two different stages using genotyping from peripheral blood. These authors identified patients carrying a rs6484711 variant A allele with a poor response to docetaxel and epirubicin in the neoadjuvant treatment. Moreover, using validation experiments, the authors identified that the rs6484711 variant A allele increased the *ABTB2* gene expression [62]. We performed a search on the PubMed database using the keywords: “Chemoresistance” AND “markers” AND “breast cancer”, and we identified 68 manuscripts published between January 2019 and November 2020. We selected only manuscripts that evaluated chemoresistance markers in BC tissue samples and the marker expression was associated with chemoresistance. Nine studies were identified, and the demographic data from these studies are shown in Table 2.

## 7. Drug Delivered Systems in Chemoresistance

Usually, traditional chemotherapeutic protocols cannot target specifically cancer cells, and due tumor heterogeneity, cancer cells may develop resistance during treatment. The use of drug delivery systems has emerged as a new alternative to revert chemoresistance [70]. Identifying the molecular pattern of chemoresistant cells, it is possible to develop nanoparticles targeting resistant cancer cells. For example, chemoresistance can be associated with the overexpression of surface markers by cancer cells. In this case, it is possible to develop nanoparticles targeting this surface marker, with the drug delivery system identifying these specific resistant cells [70]. Besides that, the use of nanoparticles can increase the drug delivery into the tumor microenvironment, overlapping the chemoresistance. Thus, in cases of chemoresistance associated with cancer stem cells and tumor-associated fibroblasts, it is possible to develop systems with high precision to delivery drugs to these cell subtypes. Among the published studies, most of them evaluated drug delivery systems focused on chemoresistance using cell line models.

Toh et al. [71] evaluated the use of nanodiamond-mitoxantrone complexes as an option for drug retention in the cell cytoplasm. One of the mechanisms associated with chemoresistance is the overexpression of cellular efflux markers (discussed in Section 4.1). Thus, the development of a drug delivery system that avoids cellular efflux represents one way to use drug delivery systems to avoid chemoresistance [71]. The nanodiamond-mitoxantrone complex (NMC) enhances the drug retention against the gradient concentration and cellular drug efflux machinery [71]. Thus, BC cells presenting chemoresistance associated with ABC transporter expression markers can be more sensitive to drugs when associated with nanodiamond particles [71]. Toh et al. [71] reported a high concentration of NMC in chemoresistant cells, demonstrating the potential of this drug delivery system to avoid this specific type of chemoresistance. Thus, the authors concluded that this system may increase BC cell sensitivity to mitoxantrone and may represent a clinical alternative for chemoresistant tumors associated with the overexpression of cellular drug efflux proteins [71].

According to Abou-El-Naga et al. [72], chemoresistant BC cells can express folic acid (FA), and drug delivery systems with an affinity for FA could represent a strategy to avoid chemoresistance. Thus, these authors, using human primary BC cell lines, developed a system based on polymeric nanoparticles encapsulating docetaxel to target FA-overexpressing cells [72]. Overall, the authors demonstrated an efficient encapsulation of docetaxel into polymeric nanoparticles and highlighted its potential to avoid cancer cell chemoresistance, maybe achieving the best results in the treatment of BC patients [72].

Among the chemoresistant phenotype, BC chemoresistance to doxorubicin has been associated with farnesyl pyrophosphate synthase overexpression [73]. The aminobisphosphonate zoledronic acid (ZA) inhibits farnesyl pyrophosphate synthase and was previously associated with a reversion of chemoresistance. Thus, ZA has been studied and clinically used to reverse cancer chemoresistance [73]. According to this concept, a research group developed a nanoparticle-based zoledronic acid-containing (NZ) drug delivery system that proved to intratumorally enhance drug delivery compared to free ZA [74,75]. On the other hands, cells with drug resistance phenotypes and cells from tumor microenvironments expressing farnesyl pyrophosphate synthase are a target of these drug delivery systems. Thus, the nanoparticle-based ZA system demonstrated the potential to target multiple chemoresistance targets, such as cancer cells and tumor microenvironments [74]. Besides that, NZ overcome doxorubicin resistance through the restoration of immunogenic cell death and increasing the doxorubicin intracellular retention [74].

Based on this previous data, is it possible to assume that drug delivery systems may represent an important strategy to avoid tumor chemoresistance, and future clinical studies are necessary to confirm the applicability of drug delivery systems in targeting chemoresistant cells.

## 8. Emerging Targets for Treatment

**Figure 4:**
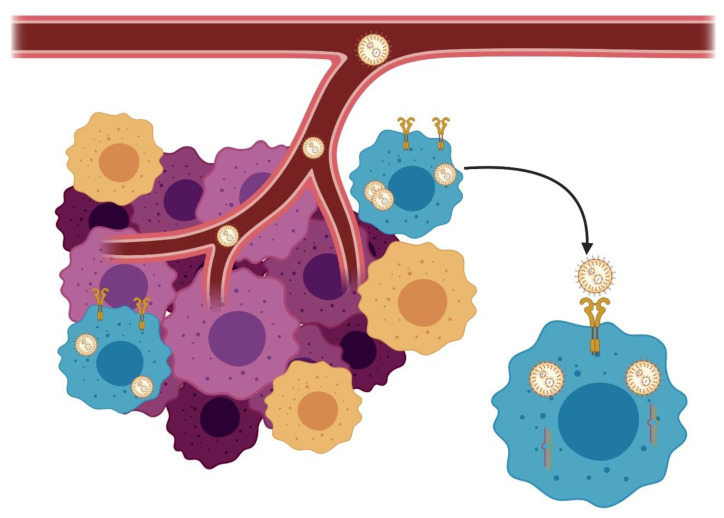
Representation of a solid lipid nanoparticle delivering small-interfering RNAs (siRNAs) in cancer cells. Through the blood vessels, nanoparticles can carry siRNA that can acts in specific BC cells, increasing the intracellular concentrations of the specific siRNA. Figure was created in BioRender (https://biorender.com/).

Due the advances on molecular oncology, in the past 20 years, we increased our knowledge regarding posttranscriptional mechanisms involved in gene regulation, including microRNAs and long noncoding RNAs [76]. Since several alterations associated with BC resistance are associated with posttranscriptional regulation, the discovery of RNA interference or small-interfering RNAs (siRNAs) brought a new therapeutic perspective [76,77]. siRNA is considered more specific than tyrosine kinase inhibitors, and the association of siRNA with drug delivery systems can revolutionize BC treatment. Regarding the drug delivery systems commercially available to siRNA transfection, we can highlight the liposomes [77]. Some strategies can be used to improve nanoparticle accumulation in cancer cells, including the coated targeting ligands highly expressed in cancer cells (i.e., HER-2). This strategy can increase nanoparticle endocytosis, increasing its concentration in target cells (Figure 4).

Some siRNA nanoparticles have been used in clinical trials of patients with solid tumors and have the potential to be applied for triple-negative BC (TNBC) patients. Some of the potential siRNA drug delivery systems previously published with clinical potential in TNBC are described in Table 3.

We performed a search on the National Institute of Health (NIH) clinical trial homepage (https://www.clinicaltrials.gov/) using the following terms: condition or disease: “Breast Cancer” and other terms: “siRNA” and “nanoparticle” to identify current or past clinical trials using nanoparticles associated with siRNA for BC treatment. It identified two previous clinical trials (Identifiers: NCT01437007 and NCT00689065), both completed. Due the potential of siRNA for the treatment of chemoresistant BC, a new search was performed in the NIH clinical trial database, modifying the condition to “Cancer” and using as the “Other terms” the words “siRNA” and “nanoparticle”. In the new search hits, 13 results were found. From these 13 results, we selected the studies performed in solid tumors that potentially could be applied to BC and identified nine clinical trials. Each trial information is available in Table 4.

## 9. Conclusions

Among the BC subtypes, TNBC represents a clinical challenging, and its chemoresistance is one of the major problems during chemotherapy. The recent increased search of epigenetic mechanisms in cancer development have increased the information regarding cancer cell chemoresistance and represent a therapeutic potential of chemoresistant BC. The use siRNA associated with different drug delivery systems can provide a new therapeutic perspective for chemoresistant TNBC.

## Figures and Tables

**Table 1:** Dysregulated microRNAs associated with chemoresistant breast cancer patients evaluated by transcriptome analysis.

MicroRNA	Expression	Fold Change
hsa-miR-195a-5p	upregulated	5.44
hsa-miR-4266	upregulated	3.45
hsa-miR-200b-3p	upregulated	3.13
hsa-miR-214-3p	upregulated	3.00
hsa-miR-107	upregulated	2.96
hsa-miR-4454	upregulated	2.88
hsa-miR-5100	upregulated	2.41
hsa-miR-23a-3p	upregulated	2.30
hsa-miR-23b-3p	upregulated	2.29
hsa-miR-16-5p	upregulated	2.09
hsa-miR-4707-5p	downregulated	0.49
hsa-miR-3656	downregulated	0.46
hsa-miR-1233-1-5p	downregulated	0.46
hsa-miR-3621	downregulated	0.44
hsa-miR-3141	downregulated	0.44
hsa-miR-489	downregulated	0.41
hsa-miR-1227-5p	downregulated	0.41
hsa-miR-1275	downregulated	0.39
hsa-miR-1268b	downregulated	0.36
hsa-miR-572	downregulated	0.30
hsa-miR-4467	downregulated	0.29
hsa-miR-4472	downregulated	0.18

**Table 2:** Information from the nine recent studies that evaluated chemoresistance markers in BC tissue samples.

Reference	Drug	Number of Petients	Markers	Expression	BC * Subtype
Rodrigues-Ferreira et al. [61]	Paclitaxel	133	ATIP3	Overexpression	
Gong et al. [62]	Epirubicin and Docetaxel	421	*ABTB2*	Overexpression	
Amri et al. [63]	Epirubicin/cyclophosphamide	6	*TCHH, MUC17, ARAP2, FLG2, ABL1, CENPF, COL6A3, DMBT1, ITGA7, PLXNA1, S100PBP, SYNE1, ZFHX4,* and *CACNA1C*	Somatic variance	Estrogen receptor-positive/HER2-negative
Jiang et al. [64]	Trastuzumab	12	NCAPG	Overexpression	HER2-positive
Chen et al. [65]	doxorubicin	20	lnc-TRDMT1-5	Overexpression	Not informed
Demir et al. [66]	doxorubicin	26	*TWIST1*	Overexpression	Not informed
Zhao et al. [67]	TEC (paclitaxel 135 ~ 175 mg/m^2^ or docetaxel 75 mg/m^2^, epirubicin 60 mg/m^2^, cyclophosphamide 600 mg/m^2^)	53	Indoleamine 2,3-dioxygenase	Overexpression	All subtypes **
Xing et al. [68]	Anthracycline-based chemotherapy	524	FKBP12	Downexpression	Luminal, HER-2 overexpressing and TNBC ***
Wang et al. [69]	CMF (cyclophosphamide + methotrexate + fluorouracil) and FEC-P (fluorouracil + epirubicin + cyclophosphamide + paclitaxel)	165	NNMT	Overexpression	All subtypes **

* BC: breast cancer. ** TNBC: triple-negative breast cancer. *** This study evaluated the BC molecular phenotypes and included all the different molecular subtypes.

**Table 3:** Small-interfering RNA (siRNA) drug delivered systems with the potential to be applied to triple-negative breast cancer. lncRNAs: long noncoding RNAs.

Target	Function	Drug Delivery System	Reference
FoxM1	Cell cycle regulator	Liposomal lipid nanoparticles	Hamurcu et al. [78]
CDK11	Cell grwoth and survival	Polyamine-based micelles	Kren et al. [79]
CDK1	Cyclin-dependent kinase	Cationic lipid-based nanoparticle made of polylactic acid and polyethylene glycol system	Liu et al. [80]
POLR2A	Catalytic component of RNA polymerase II	pH-activated nanoparticles	Xu et al. [81]
AKT1	Regulator of mTOR signaling pathway	Inorganic amorphous calcium carbonate (ACC) hybrid nanospheres functionalized with CaIP6 (ACC/CaIP6) nanoparticles	Zhou et al. [82]
onco-lncRNAs	Influences gene signature	1-aminoethylimino[bis(N-oleoylcysteinyl-aminoethyl)propionamide]- polyethylene glycol-RGD/siRNA nanoparticles	Vaidya et al. [83]

**Table 4:** Information from the nine selected clinical trials identified in the National Institute of Health (NIH) clinical trial database.

Status	Brief Description	Interventions/Treatment	Phase	Study Title
Active, not recruiting	This phase I trial studies the side effects and best dose of APN401 in treating patients with pancreatic cancer, colorectal cancer or other solid tumors that have spread to other places in the body or have come back. APN401 may stop the growth of tumor cells by blocking some of the enzymes needed for cell growth.	siRNA-transfected Peripheral Blood Mononuclear Cells APN401	I	APN401 in Treating Patients With Recurrent or Metastatic Pancreatic Cancer, Colorectal Cancer, or Other Solid Tumors That Cannot Be Removed by Surgery
Completed	This phase I trial studies the side effects and best dose of small-interfering ribonucleic acid (siRNA)-transfected peripheral blood mononuclear cells APN401 (APN401) in treating patients with melanoma, kidney or pancreatic cancer or other solid tumors that have spread to other parts of the body or that cannot be removed by surgery. There are factors in immune cells in the blood that inhibit their ability to kill cancers. Treating white blood cells with one of these factors in the laboratory may help the white blood cells kill more cancer cells when they are put back in the body.	Biological: siRNA-transfected peripheral blood mononuclear cells APN401	I	APN401 in Treating Patients With Melanoma, Kidney Cancer, Pancreatic Cancer, or Other Solid Tumors That Are Metastatic or Cannot Be Removed By Surgery
Not yet recruiting	This phase I trial studies the best dose and side effects of mesenchymal stromal cell-derived exosomes with KrasG12D siRNA (iExosomes) in treating participants with pancreatic cancer with a KrasG12D mutation that has spread to other places in the body. iExosomes may work better at treating pancreatic cancer.	Mesenchymal Stromal Cells-derived Exosomes with KRAS G12D siRNA	I	iExosomes in Treating Participants With Metastatic Pancreas Cancer With KrasG12D Mutation
Completed	Cancer in the liver can start in the liver (e.g., primary liver cancer or hepatocellular cancer) or spread to the liver from cancers in other parts of the body (e.g., colon, pancreas, gastric, breast, ovarian, esophageal cancers and cancer with metastases to the liver). People who have tumors that can be removed by surgery live longer than those whose cancer cannot be removed. Chemotherapy can shrink some tumors in the liver, which also helps people to live longer, and sometimes, chemotherapy can shrink tumors enough that they can be removed by surgery. However, most chemotherapy drugs do not work well on tumors in the liver. In this study, we are testing a new drug, TKM-080301, given directly into the cancer blood supply in the liver circulation to see if it will cause tumors to shrink.	TKM-080301	I	TKM 080301 for Primary or Secondary Liver Cancer
Completed	Phase I: This study is designed to investigate the safety of a siG12D LODER (Local Drug EluteR) in patients diagnosed with adenocarcinoma of the pancreas. The primary endpoint is to assess the efficacy of the siG12D LODER and local distribution in nonoperable patients by histopathology measurements and local distribution by RNA analysis.	siG12D LODER	I	Phase I - Escalating Dose Study of siG12D LODER (Local Drug EluteR) in Patients With Locally Advanced Adenocarcinoma of the Pancreas, and a Single Dose Study of siG12D LODER (Local Drug EluteR) in Patients With Non-operable Adenocarcinoma of the Pancreas
Terminated	The purpose of this study is to assess the safety and tolerability of the investigational anticancer drug DCR-MYC. DCR-MYC is a novel synthetic double-stranded RNA in a stable lipid particle suspension that targets the oncogene MYC. MYC oncogene activation is important to the growth of many hematologic and solid tumor malignancies. In this study, the sponsor proposes to study DCR-MYC and its ability to inhibit MYC and thereby inhibit cancer cell growth.	DCR-MYC	I	Phase I, Multicenter, Dose Escalation Study of DCR-MYC in Patients With Solid Tumors, Multiple Myeloma, or Lymphoma
Unknown	In this Phase II study, a dose of 2.8 mg (eight 0.35-mg siG12D-LODERs) will be administered in 12-week cycles to patients with unresectable locally advanced pancreatic cancer combined with chemotherapy treatment.	siG12D-LODER	II	A Phase 2 Study of siG12D LODER in Combination With Chemotherapy in Patients With Locally Advanced Pancreatic Cancer
Recruiting	This phase I trial studies the side effects and best dose of EphA2 siRNA in treating patients with solid tumors that have spread to other places in the body and usually cannot be cured or controlled with treatment (advanced) or have come back after a period of improvement (recurrent). EphA2-targeting 1,2-dioleoyl-sn-glycero-3-phosphatidylcholine -encapsulated siRNA may slow the growth of tumor cells by shutting down the activity of a gene that causes tumor growth.	EphA2-targeting DOPC-encapsulated siRNA	I	EphA2 siRNA in Treating Patients With Advanced or Recurrent Solid Tumors
Terminated	The purpose of this study is to assess the safety and tolerability of the investigational anticancer drug DCR-MYC. DCR-MYC is a novel synthetic double-stranded RNA in a stable lipid particle suspension that targets the oncogene MYC. MYC oncogene activation is important to the growth of many hematologic and solid tumor malignancies. In this study, the sponsor proposes to study DCR-MYC and its ability to inhibit MYC and thereby inhibit cancer cell growth.	DCR-MYC	II	Phase Ib/2, Multicenter, Dose Escalation Study of DCR-MYC in Patients With Hepatocellular Carcinoma

Information retrieved from: https://www.clinicaltrials.gov/.
